# Machine stability and dosimetry for ultra‐high dose rate FLASH radiotherapy human clinical protocol

**DOI:** 10.1002/acm2.70102

**Published:** 2025-04-10

**Authors:** Patrik Gonçalves Jorge, Reiner Geyer, Rémy Kinj, Luis Schiappacasse, Wendy Jeanneret‐Sozzi, Jean Bourhis, Fernanda Herrera, François Bochud, Claude Bailat, Raphaël Moeckli

**Affiliations:** ^1^ Institute of Radiation Physics Lausanne University Hospital and Lausanne University Lausanne Switzerland; ^2^ Department of Radiation Oncology Lausanne University Hospital and Lausanne University Lausanne Switzerland

**Keywords:** dosimetry, FLASH, monitoring, reproducibility, stability, UHDR, ultra‐high dose rate

## Abstract

**Background:**

The FLASH effect, induced by ultra‐high dose rate (UHDR) irradiations, offers the potential to spare normal tissue while effectively treating tumors. It is important to achieve precise and accurate dose delivery and to establish reliable detector systems, particularly for clinical trials needed to help the clinical transfer of FLASH‐Radiotherapy (FLASH‐RT). However, the use of monitoring chambers with UHDR beams is presently limited, leading to the reliance on passive dosimetry and machine stability.

**Purpose:**

This study aimed to investigate the energy and output stability of a UHDR Mobetron (IntraOp, USA) and to compare it with its conventional dose rate (CDR) mode. Furthermore, we assessed the dosimetric accuracy of a human clinical protocol for FLASH‐RT.

**Methods:**

Over a 26‐month duration, we assessed the short‐ and long‐term stability of the output and energy of the Mobetron system. Daily checks were conducted for 9 MeV CDR and UHDR. In parallel, the IMPulse clinical trial involving patients with skin metastases from melanoma was initiated. Prescription doses ranging from 22 to 28 Gy were administered. Pre‐, post‐, and *in vivo* dosimetry using alanine and thermoluminescent dosimeters (TLDs) was performed and compared to the prescription doses.

**Results:**

Short‐term output fluctuations remained below 0.6 % and 1 % for 9 MeV CDR and UHDR, respectively. Long‐term output fluctuations were within 2 % and the long‐term energy fluctuations were below 2 mm (R_50_) for both modes. The delivered doses of the IMPulse trial showed deviations below 4 % compared to prescription doses for all patients.

**Conclusions:**

The Mobetron system demonstrated favorable short‐ and long‐term stability. There was a good agreement between the prescribed and the measured dose for the clinical IMPulse trial. The stability of this UHDR machine allows us to effectively conduct human clinical protocols as well as preclinical experiments, even in the absence of a real‐time monitoring system.

## 1 INTRODUCTION

In the last decades, ultra‐high dose rate (UHDR) irradiations have shown the ability to spare normal tissue while effectively eliminating tumors, holding the potential for improving the therapeutic index in radiotherapy (RT). This phenomenon, known as the FLASH effect, has garnered significant interest in both the radiobiology community and clinical practice, with efforts already underway for its clinical translation.^1^, ^2^, ^3^, ^4^, ^5^

A phase I trial, published in 2019, focused on treating superficial squamous cell carcinoma (SCC) in feline patients with curative intent.^6^ This study escalated doses from 26 to 41 Gy in a single fraction using 4.5 MeV electrons at UHDR, demonstrating the efficacy and safety of FLASH‐RT. Subsequent to these encouraging results, a phase III trial was initiated, comparing a single fraction of 30 Gy with 5.5 MeV electrons at UHDR against a fractionated standard regimen with 6, 9, or 12 MeV electrons at conventional dose rate (CDR).^7^ This trial showed an equivalent long‐term tumor control outcome in both arms. Concurrently, a trial involving canine patients across various cancer types explored doses ranging from 15 to 35 Gy using a 10 MeV electron beam at UHDR, with no observed short‐term toxicity.^8^ However, these two latter studies showed long‐term toxicities, probably due to overdosage. In addition, the treatment of the first human patient with a cutaneous lymphoma was successfully conducted, employing a single fraction of 15 Gy with a 5.5 MeV electron beam at UHDR.^9^ While the majority of the published data focuses on electron irradiation, only one trial to date has reported the use of UHDR proton beam (230 MeV) for treating extremity bone metastases with a single‐transmission beam and a single dose of 8 Gy.^10^

The UHDR beams used for preclinical and clinical studies present technological challenges, primarily due to the significantly higher dose rates employed compared to standard clinical practice. Unlike CDR RT, where the output is consistently controlled using two independent monitoring chambers, UHDR beams saturate commercially available monitoring chambers and they are not able to react quickly enough, rendering them ineffective in the context of FLASH‐RT.^11^

Should uncertainties exist regarding dosimetry and its stability, it poses a risk to the integrity of experiments and protocols, potentially resulting in erroneous conclusions regarding the FLASH effect. The absence of short‐term dosimetric stability could obscure the observation of the FLASH effect, as it risks being masked by the dispersion and inconsistency of the data, compromising the ability to discern the FLASH effect accurately and undermining the validity of research findings. Without long‐term dosimetric stability, measurements in a given condition are only valid for a limited amount of time, forcing frequent beam parameter adjustments and increasing workload. As a result, the short‐ and long‐term stability of the UHDR machines becomes a critical factor in successfully preparing and completing irradiation at UHDR.^12^

The Oriatron eRT6 (PMB Alcen, France) demonstrated notable stability, with short‐term stability reported to be less than 1 % and long‐term stability at 4.1 % over 20 months.^13^ A modified Elekta system only reported short‐term stability of 1 %–4 % valid for the first 10 min, but it could reach 11 % after 10 min. No long‐term data was available for that device.^14^ No stability data was found for other electron UHDR devices. This scarcity of stability‐focused studies underscores the need for comprehensive assessments of the stability of UHDR systems to ensure accurate and reliable dose delivery in clinical and preclinical applications.

The Mobetron is a medical device developed by IntraOp Medical Corporation specifically designed for intraoperative electron radiation therapy (IOERT), offering a range of beam energies from 4 MeV to 12 MeV suitable for various clinical applications. These high‐energy beams are employed for deeper‐seated tumors and are able to deliver high doses directly to the tumor bed during surgery.^15^ An upgrade of the Mobetron has enabled it to perform UHDR irradiations and was successfully commissioned in the context of FLASH preclinical animal experiments and FLASH human clinical trials.^16^

Studies of the FLASH effect with the upgraded UHDR Mobetron at two independent institutions have yielded comparable results with studies focusing on acute effects after total abdominal irradiation in mice.^17^ Notably, this machine is also utilized to perform the first‐in‐human FLASH‐RT clinical trial IMPulse with 9 MeV electrons targeting skin metastases from melanoma.^18^ In addition, a randomized clinical phase II study comparing curative FLASH‐RT versus CDR RT for cutaneous basal cell carcinoma and SCC (LANCE) has been initiated with the same machine.^19^, ^20^

In this study, we investigated the short‐ and long‐term output and energy stability of an upgraded UHDR Mobetron over a 26‐month period. Within the context of the IMPulse clinical trial, we validated our dosimetric procedure by performing pre‐, post‐, and *in vivo* dosimetry, comparing the measured doses with the prescribed doses for all patients enrolled in the trial.^18^

## 2 MATERIAL AND METHODS

### 2.1 Irradiation device and setup

For our study, we assessed an upgraded version of the Mobetron with a 9 MeV electron beam commissioned to operate in both CDR and UHDR regimes.^16^ While the upgraded Mobetron was also commissioned for a 6 MeV UHDR electron beam, our focus remained on the 9 MeV CDR and UHDR modalities given that most of our biological experiments are performed at the same beam energy (9 MeV). We chose this approach to monitor the machine during the protocol and to establish a reference point for our investigations.

It should be noted that the device used for the two trials was not equipped with dual beam current transformers (BCTs) in contrast with other units that were sold by IntraOp Inc. after the start of the IMPulse and LANCE trials.^21^ Henceforth, for simplicity, we refer to this upgraded Mobetron capable of performing both UHDR and CDR irradiations as “Mobetron”.

In CDR mode, the beam parameters are fixed with a reference pulse repetition frequency (PRF) of 30 Hz and a reference pulse width (PW) of 1.2 µs, resulting in a dose per pulse of about 10 mGy and a reference dose rate of approximately 1000 Monitor Units (MU) per minute controlled by two independent monitoring chambers. In this mode, users can only adjust the number of MUs to match the planned dose.

In UHDR mode, users have greater flexibility and the beam parameters can be set within the acceptable operating limits of the major system components. This versatility enables users to control the beam structure, such as the number of pulses (N_p_), the PW, and the PRF. The resulting dose per pulse can reach over 3 Gy in this mode. Additional details regarding the Mobetron and its characteristics can be found elsewhere.^16^

### 2.2 Output and energy stability

Daily and monthly controls were carried out over a period of 26 months with both 9 MeV CDR and UHDR beams to assess output and energy stability over time. For these tests, a fixed set of beam parameters was selected as described in Table 1.

**TABLE 1:** Beam parameters for CDR and UHDR beams for daily and monthly controls.

Beam	MU/N_p_	PRF (Hz)	PW (µs)
9 MeV CDR	500	30	1.2
9 MeV UHDR	2	90	4.0

Abbreviations: CDR, conventional dose rate; MU, monitor units; N_p_, number of pulses; PRF, pulse repetition frequency; PW, pulse width; UHDR, ultra‐high dose rate.

Measurements were performed in RW3 solid water slabs (PTW‐Freiburg GmbH, Freiburg, Germany) with an Advanced Markus ionization chamber (PTW‐Freiburg GmbH, Freiburg, Germany). For output measurements, the ionization chamber was placed in the solid water phantom at 1 cm depth with a Source‐Axis‐Distance (SAD) of 100 cm. Additional measurements were performed at 3.5 cm depth and 100 cm SAD. The measurements at 1 cm depth provided information about the output. The ratio of measurements at 1 cm and 3.5 cm depth was used to monitor energy stability.

Three measurements were performed daily and 10 monthly at each depth with an Advanced Markus ionization chamber associated with a PTW UNIDOS electrometer (PTW‐Freiburg GmbH, Freiburg, Germany). The standard uncertainty on dose measurements with the ionization chamber was 2.8 % (*k *= 1) as saturation correction factors were applied.^22^ In this setup, the dose per pulse was low enough (about 0.3 Gy) to minimize the impact of the saturation effect and the estimated ion collection efficiency was about 90 % at UHDR, resulting in small saturation correction factors.^22^ Regardless, the absolute value of the dose is unimportant, as we conducted relative measurements under consistent conditions.

The Standard Deviation (SD) over the 10 consecutive measurements from the monthly controls provided insight into the short‐term stability of the output, while the difference between the average of the daily values and a reference output value (established during commissioning) assessed long‐term output stability. The short‐term stability of the energy was assessed by the SD over the 10 consecutive ratios from monthly controls, while long‐term energy stability was evaluated by comparing a reference ratio value (established during commissioning) with the average daily ratio.

According to AAPM TG142 recommendations, the depth on the percentage depth dose (PDD) curve at which the PDD attains a value of 50 % (called R_50_) is recommended to be used to follow the stability of the energy, with variations not exceeding 2 mm.^23^, ^24^ To align with these recommendations, we converted our energy indicator variation to R_50_ variation assuming a constant slope of the PDD around R_50_.

The output/energy monitoring was interrupted twice due to machine malfunctions related to the solid‐state modulator (SSM), resulting in a temporary suspension of measurements and patient recruitement. However, after manufacturer intervention, the machine was restored to its initial state and measurements resumed.

### 2.3 IMPulse trial dosimetric monitoring

The IMPulse trial is a single‐center phase I clinical trial (three‐by‐three dose escalation study) evaluating the efficacy and safety of FLASH‐RT in patients with skin melanoma metastases.^18^ The primary endpoint of the study was to determine the maximal tolerated dose (MTD) or recommended phase II dose (RP2D) for FLASH‐RT that can durably control skin melanoma metastases, without significant side effects. The secondary endpoint focuses on the effect of FLASH‐RT on cutaneous relief of symptoms (pain, hemorrhage, skin ulceration), local response of metastases, and acute and late side effects in the radiation field. The study consisted of two experimental arms based on lesion volumes: small (< 30 cc) and large (> 30 cc).

The dose was prescribed at the skin level with treatment doses ranging from 22 to 34 Gy in 2 Gy increments. Our reported data focuses on dosimetry for doses between 22 to 28 Gy. Each dose level included three treated lesions. However, there are exceptions in the reported data: for the 22 Gy level, a large lesion was included; for the 24 Gy level, one patient with two lesions was excluded from the study due to the inability to perform follow‐up; and for the 28 Gy level, data for one lesion is still pending to complete the dose level.

For this trial, the 9 MeV UHDR beam was employed, with N_p_ fixed at 10 and PRF set to 90 Hz, resulting in PWs ranging from approximately 2.3  up to 3 µs and doses‐per‐pulse ranging from 2.2  up to 2.8 Gy with an average dose rate of 220–280 Gy/s depending on the delivered dose. In other words, the N_p_ and the total treatment time remained constant and only the dose per pulse was changed for each dose level. The target volume, comprising the visible skin lesion with a 5 mm margin, was delineated to ensure adequate coverage of the tumor while minimizing irradiation of surrounding healthy tissue. Circular collimators with diameters ranging from 2 to 5 cm^16^ were selected based on the size of the target volume, effectively delineating the treatment field and optimizing radiation delivery.

The dosimetric procedures for UHDR irradiations followed established protocols using alanine pellets and thermoluminescent dosimeters (TLDs).^25^ We used alanine pellets (Harwell Dosimeters Ltd, Oxfordshire, UK) with dimensions of 4.8 mm diameter and 2.8 mm thickness. They were analyzed using a Bruker e‐scan EPR spectrometer (Bruker Corporation, Billerica, Massachusetts, USA), with an optimized reading protocol to achieve a 2.2 % uncertainty (*k *= 1).^26^ LiF‐100 TLDs (Thermo Fisher, USA) of dimensions 3.2 × 3.2 × 0.9 mm^3^, individually calibrated in terms of absorbed dose to water using a Co‐60 unit and a correction factor for electrons, were also utilized with an uncertainty around 4 % (*k *= 1) for electron measurements.^13^

To verify the dosimetric protocol and better assess the machine's response for each treatment, pre‐and post‐treatment dosimetric controls were performed with three TLDs and three alanine pellets. The dosimeters were inserted into a homemade holder that could be placed into the slot reserved for the ionization chamber in RW3 solid water plates.^25^ The dosimeter effective depth was 3.4  and 1.5 mm for the alanine pellet and TLD respectively. Surface doses were extrapolated from the measured dose based on the PDD of the applicator.^16^ In addition, *in vivo* dosimetry was realized with an alanine pellet located in the treatment area at the surface of the skin. Pre‐ and post‐dosimetric results were analyzed together and compared to the prescribed dose while *in vivo* measured results were analyzed separately. The maximum accepted dose deviation for the IMPulse trial was fixed to 5 %.^18^

## 3 RESULTS

### 3.1 Output stability

**FIGURE 1:**
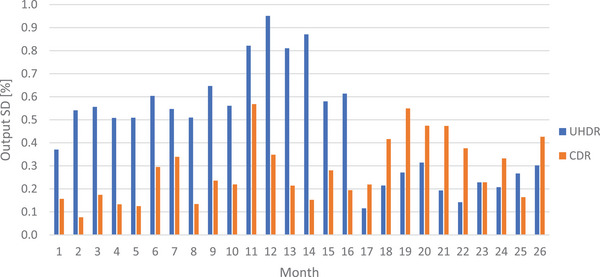
Output SD illustrating the short‐term output fluctuations over consecutive irradiations for both beam modalities from monthly controls. SD, standard deviation.

Figure 1 illustrates the output short‐term fluctuations for CDR and UHDR beams. Throughout the evaluation period, the CDR mode exhibited consistent short‐term output stability, with fluctuations ranging between 0.1 % and 0.6 % and averaging at 0.3 %. The UHDR mode displayed larger short‐term fluctuations, ranging from 0.1 % to 1.0 %. Notably, a significant decrease in output SD was observed from month 17 onwards (July 2023), following machine component modifications (machine maintenance) and tuning after breakdown. Before this intervention, the mean output SD was 0.6 %, which decreased to 0.2 % thereafter.

**FIGURE 2:**
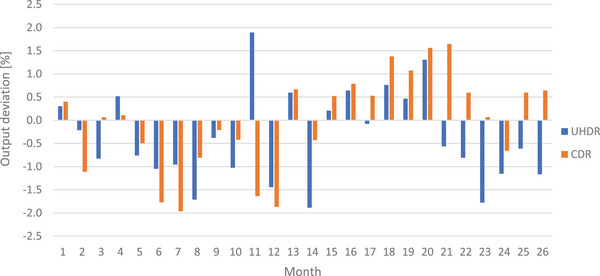
Long‐term output fluctuations for both beam modalities from daily controls.

Figure 2 presents the long‐term output fluctuations for CDR and UHDR beams. For clarity, only the average output shift for each month is displayed. Comparable average output differences were observed between CDR (0.8 %) and UHDR (0.9 %) modes. Additionally, maximum absolute daily output fluctuations remained below 2.0 % for CDR and 2.6 % for UHDR.

### 3.2 Energy stability

The energy short‐term stability for both beam modalities is provided in the supplementary data (Figure S1). Both beam modalities show comparable R_50_ fluctuations remaining below 0.5 mm.

**FIGURE 3:**
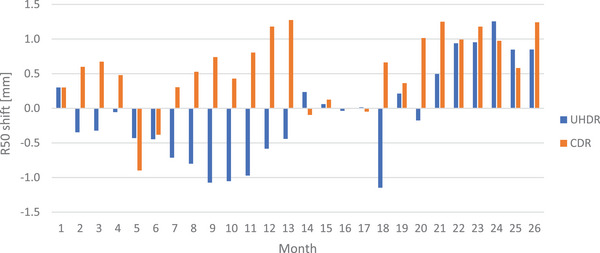
Long‐term energy fluctuations according to R_50_ for both beam modalities from daily controls.

Figure 3 illustrates the long‐term energy shifts in terms of R_50_ for both CDR and UHDR beam modalities. Only the average output shift for each month is depicted. The absolute average R_50_ shifts were comparable between CDR (0.7 mm) and UHDR (0.6 mm) modes, with maximum daily R_50_ shifts of 1.4 mm for CDR and 1.8 mm for UHDR.

### 3.3 IMPulse dosimetric monitoring

**FIGURE 4:**
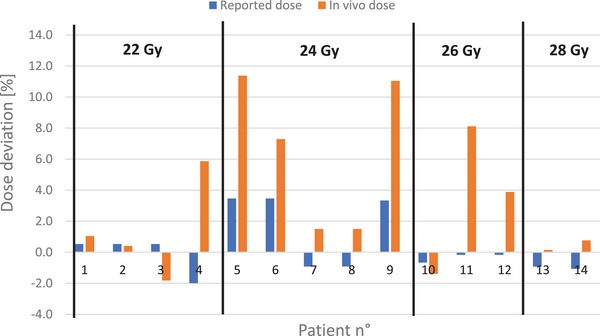
Mean dose deviation with respect to the prescription dose and *in vivo* dosimetry for each lesion treated in the IMPULSE protocol.

Figure 4 displays the dose deviations between the prescription dose and the reported dose based on pre‐and post‐treatment dosimetry as well as *in vivo* results. Pre‐ and post‐treatment dose deviations remained below 4 % for all treatments, with an average deviation of 1.3 %. Regarding *in vivo* measurements, twelve out of fourteen measurements indicated higher doses than the prescription dose, with deviations ranging from −0.8 % to +11.4 %. On average, measured doses were 3.7 % higher than the prescription dose.

## 4 DISCUSSION

We investigated the energy and output stability of an upgraded UHDR Mobetron device, comparing its performance with the CDR mode, controlled by monitoring chambers. Over more than two years, we conducted over 1000 measurements to thoroughly characterize the stability of this UHDR device capable of performing FLASH‐RT. To our knowledge, this study is the first to explore output/energy stability monitoring of a UHDR device over such an extended period. In addition, we evaluated the dosimetry related to the IMPulse protocol. Our findings underscore the crucial link between accurate dosimetry and robust output/energy stability, validating our dosimetric strategy and presenting the possible and effective use of a UHDR device without any monitoring system.

For the Mobetron, short‐term output monitoring revealed fluctuations for the CDR beam throughout the measurement period of 0.3 % on average. Such stability was expected due to the CDR beam's control by two independent monitoring chambers. For the UHDR beam, stability improved significantly following machine component modifications (machine maintenance) and tuning after breakdown, reducing the output SD approximately by a factor of two. This improvement was not only due to component replacement but also benefited from the enhanced expertise of the engineering team, reflecting a learning curve in the machine tuning expertise. Since then, short‐term output fluctuations are comparable between UHDR and CDR modalities, with similar ranges of fluctuations within acceptable limits (SD < 0.6 %).

Given the high dose per pulse (about 3 Gy per pulse), only a few pulses are required to deliver the prescribed dose, which could lead to significant dose deviations if the planned N_p_ is not accurately delivered (in the case of the IMPulse protocol, 10 % of the total dose is delivered per pulse). Out of over 10 000 measurements conducted on our device in recent years by various operators, the measured dose was consistent except in three cases, where the results were compatible (but not demonstrated) with a missing pulse. No measured dose was compatible with an additional unwanted pulse.

In the event of a missing or additional pulse, BCTs would detect the inconsistency between the number of desired and delivered pulses but would still be unable to control the beam quickly enough and react to compensate (in the case of a missing pulse). For the clinical transfer of FLASH‐RT, a monitoring device capable of real‐time output measurement and beam control is necessary.

Future advancements in real‐time monitoring for FLASH‐RT are expected to benefit from innovations in detector technology and adaptive control systems. Emerging alternatives, such as ultra‐thin silicon detectors and diamond‐based sensors, offer superior sensitivity and spatial resolution while minimizing recombination effects.^27^ Additionally, multi‐gap in‐transmission ionization chambers present a promising solution for mitigating recombination, ensuring more accurate charge collection.^28^ Passive resonant cavities, already employed in high‐energy physics, could further enhance real‐time beam current measurements with improved precision.^29^ Additionally, as previously mentioned, integrating BCTs into the monitoring system can enhance charge measurement accuracy and reproducibility.^11^, ^30^

Beyond real‐time monitoring, a major challenge in UHDR beam delivery is achieving precise pulse‐by‐pulse control to ensure the prescribed dose is met within extremely short time frames. Certain electronic systems, such as ICTs, already enable ultra‐fast signal readout and processing in the millisecond range. Their capability to provide real‐time data on beam output and energy has been demonstrated, suggesting their potential role in actively controlling UHDR beams in the future.^21^, ^30^

Short‐term stability is crucial for biological samples irradiation and, more importantly, patient treatment, because no monitoring device is capable of measuring and controlling the beam quickly enough yet. Therefore, short‐term stability allows us to conduct a “sandwich” measurement—consisting of one measurement before and one after—providing a representative assessment of the delivered dose in between. This method serves as a safeguard against potential discrepancies or errors in dose delivery, ensuring reasonable accuracy and reliability of our dosimetric assessments, considering the probability of encountering a missing or additional pulse.

Long‐term output and energy fluctuations, as illustrated in Figures 2 and 3, also demonstrated comparable performance between UHDR and CDR. The average differences in output and R_50_ shifts between the two modalities are well within tolerances, indicating that UHDR maintains stability over extended periods of time similar to CDR. Moreover, observations following brief machine inactivity periods (several days) — during which no measurements, patient treatments, or experiments are conducted—indicate that upon restarting the machine, dosimetric measurements remain within tolerances without any need for additional tuning or specific warm‐up. This observation underscores that the stability of dosimetry measurements is not contingent upon the regular utilization of the machine.

As the maximum accepted dose deviation for the protocol IMPulse was fixed to 5 %, reported doses remained within acceptable tolerances for all patients (Figure 4), validating our dosimetric strategy with the Mobetron and instilling confidence in our UHDR device for future FLASH‐RT clinical applications.

Regarding *in vivo* dosimetry, deviations in dose measurements relative to the prescribed dose can stem from various sources owing to the inherent uncertainties in such measurements. These uncertainties may arise from factors such as the non‐uniformity of the dose profile with smaller field sizes (the field size at 90 % isodose is 1.6, 2.0, 3.9, 5.0, and 6 cm for the 2, 3, 4, and 5 cm collimators respectively^16^), patient movement during treatment, variability in setup conditions, and the shape and curvature of the lesion being treated, as we prepare dosimetry in a fixed and stable configuration. Additional measurements were performed with alanine dosimeters inserted 5 mm inside the collimator (closer to the source) and were compared to surface measurements. Notably, these measurements showed a dose increase of 5 % associated with a 5 mm shift inside the collimator. Moreover, as the *in vivo* dosimeters were not always centered with respect to the collimator, dose deviations could also come from the heterogeneity of the dose profile at the surface. As an example, dose profiles with the 3 cm collimator show a non‐uniform dose distribution with fluctuations up to 5 % within the collimator diameter at the surface level. These uncertainties, coupled with the complexity of the treatment setup (surface not flat close to a half sphere), further complicate the interpretation of *in vivo* dose measurements. However, *in vivo* dosimetry indirectly confirmed the accuracy of our dosimetric protocol in several cases, with no unexpected discrepancies detected, largely due to the implementation of our “sandwich” measurement approach.

## 5 CONCLUSION

We investigated the energy and output stability of an upgraded UHDR Mobetron device and compared them with the CDR mode. Through experimentation spanning approximately two years, we provided comprehensive insights into its stability for FLASH‐RT applications, crucial for ensuring accurate dosimetry and safe administration of treatment in FLASH‐RT applications. In addition, the evaluation of the delivered dose in the context of the IMPulse protocol was presented.

Our results emphasized the strong relationship between robust output/energy stability and precise dosimetry, validating the effectiveness of our dosimetric strategy and affirming the possible utilization of UHDR devices even in the absence of monitoring systems. We demonstrated comparable short‐term and long‐term output and energy stability between the UHDR and CDR modalities, highlighting the reliability of our UHDR device over extended durations. The observed short‐term stability facilitates accurate dosimetry and mitigates potential errors in dose delivery, while the parity in long‐term stability further underscores the consistent dose delivery capability, essential for maintaining treatment efficacy over time. Furthermore, the *in vivo* dosimeters served as valuable tools in indirectly confirming the consistency of the delivered dose with the prescription dose.

## AUTHOR CONTRIBUTIONS

**Patrik Gonçalves Jorge**: Conceptualization; data curation; formal analysis; investigation; methodology; resources; software; writing—original draft; writing—review and editing. **Reiner Geyer**: Data curation; formal analysis; methodology; validation; writing—review and editing. **Rémy Kinj**: Validation; writing—review and editing. **Luis Schiappacasse**: Validation; writing—review and editing. **Wendy Jeanneret‐Sozzi**: Validation; writing—review and editing. **Jean Bourhis**: Validation; writing—review and editing. **Fernanda Herrera**: Validation; writing—review and editing. **François Bochud**: Conceptualization; validation; writing ‐ review and editing. **Claude Bailat**: Conceptualization; formal analysis; methodology; validation; writing—review and editing. **Raphael Moeckli**: Conceptualization; methodology; project administration; supervision; validation; writing—original draft; writing—review and editing.

## CONFLICT OF INTEREST STATEMENT

This research has been partially funded by IntraOp Medical Corporation.

## Supporting information



Supporting Information
