# Supplementary material for: Machine stability and dosimetry for ultra‐high dose rate FLASH radiotherapy human clinical protocol
Source: J Appl Clin Med Phys. 2025 Apr 10;26(6):e70102. doi: 10.1002/acm2.70102 (PMC12148758; doi:10.1002/acm2.70102)
Supplement: Supplementary file 1 — Supporting Information [file ACM2-26-e70102-s001.docx]

Supplementary Figure 1: energy SD illustrating the short-term energy fluctuations over consecutive irradiations for both beam modalities from monthly controls.
